# Fibrocyte localisation to the ASM bundle in asthma: bidirectional effects on cell phenotype and behaviour

**DOI:** 10.1002/cti2.1205

**Published:** 2020-11-13

**Authors:** Ruth Saunders, Davinder Kaur, Dhananjay Desai, Rachid Berair, Latifa Chachi, Richard D Thompson, Salman H Siddiqui, Christopher E Brightling

**Affiliations:** ^1^ Department of Respiratory Sciences Institute for Lung Health University of Leicester Leicester UK; ^2^ University Hospitals Birmingham NHS Foundation Trust Birmingham UK; ^3^ Present address: University Hospitals Coventry & Warwickshire NHS Trust Coventry UK; ^4^ Present address: The Royal Wolverhampton NHS Trust Wolverhampton UK

**Keywords:** airway hyper‐responsiveness, airway remodelling, airway smooth muscle, asthma, fibrocytes

## Abstract

**Objectives:**

Airway hyper‐responsiveness and persistent airflow obstruction contribute to asthma pathogenesis and symptoms, due in part to airway smooth muscle (ASM) hypercontractility and increased ASM mass. Fibrocytes have been shown to localise to the ASM in asthma however it is not known whether fibrocytes localise to the ASM in nonasthmatic eosinophilic bronchitis (NAEB) and chronic obstructive pulmonary disease (COPD). In addition, the potential consequences of fibrocyte localisation to ASM as regards asthma pathophysiology has not been widely studied.

**Methods:**

Fibrocytes and proliferating cells were enumerated in ASM in bronchial tissue using immunohistochemistry. The effects of primary ASM and fibrocytes upon each other in terms of phenotype and behaviour following co‐culture were investigated by assessing cell number, size, apoptotic status, phenotype and contractility in *in vitro* cell‐based assays.

**Results:**

Increased fibrocyte number in the ASM was observed in asthma versus NAEB, but not NAEB and COPD versus controls, and confirmed in asthma versus controls. ASM proliferation was not detectably different in asthmatics versus healthy controls *in vivo*. No difference in proliferation, apoptotic status or size of ASM was seen following culture with/without fibrocytes. Following co‐culture with ASM from asthmatics versus nonasthmatics, fibrocyte smooth muscle marker expression and collagen gel contraction were greater. Following co‐culture, fibrocyte CD14 expression was restored with the potential to contribute to asthma pathogenesis via monocyte‐mediated processes dependent on the inflammatory milieu.

**Conclusion:**

Further understanding of mechanisms of fibrocyte recruitment to and/or differentiation within the ASM may identify novel therapeutic targets to modulate ASM dysfunction in asthma.

## Graphical abstract

Fibrocyte recruitment to the airway smooth muscle (ASM) is a feature of asthma but not chronic obstructive pulmonary disease or nonasthmatic eosinophilic bronchitis. Our *in vitro* data show that culture with ASM from asthmatics maintains a muscle‐like phenotype in fibrocytes and augments contractility. In addition, fibrocytes may retain the ability to participate in monocyte‐mediated processes, dependent on the composition of the local inflammatory milieu. Further understanding of mechanisms of fibrocyte recruitment to and/or differentiation within the ASM may identify novel therapeutic targets to modulate ASM dysfunction in asthma.

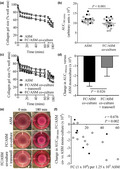

## Introduction

Asthma, a major cause of morbidity and mortality worldwide, continues to rise in prevalence.^1^ Several treatments are available/in late development for T2^HIGH^ asthma (predominantly eosinophilic with high T2 cytokines).^1^, ^2^, ^3^ However, ~50% of asthmatics are T2^LOW^ (noneosinophilic with/without neutrophilia) and T2^HIGH^ therapies do not completely abrogate symptoms in all patients.^2^, ^4^, ^5^ Airway hyper‐responsiveness (AHR) and persistent airflow obstruction contribute to asthma pathogenesis and symptoms in both T2^HIGH^ and T2^LOW^ asthma, due in part to airway smooth muscle (ASM) hypercontractility and increased ASM mass.^6^, ^7^, ^8^

Increasing evidence supports the notion that increased ASM mass in asthma is because of recruitment of ASM cells/progenitors to the ASM from the circulation/elsewhere in the tissue.^8^ Indeed, we have previously demonstrated that fibrocyte numbers are increased in the circulation, lamina propria and ASM of asthmatics versus healthy controls, that ASM‐derived mediators stimulate fibrocyte migration and that reduced ASM mass following fevipiprant treatment correlates with reduced fibrocyte number in the lamina propria.^9^, ^10^ However, the consequences of fibrocyte localisation to ASM in asthma pathophysiology have not been widely studied.

We hypothesised that, following recruitment to the ASM, fibrocytes become more smooth muscle‐like because of ASM‐derived factors with the potential to contribute to increased ASM mass and ASM hypercontractility in asthma. To test this hypothesis, we enumerated fibrocytes in bronchial tissue in asthma compared to nonasthmatic eosinophilic bronchitis (NAEB), chronic obstructive pulmonary disease (COPD) and healthy controls and investigated the effects of ASM/fibrocytes upon each other in terms of phenotype and behaviour using primary cultures.

## Results

### Fibrocytes localise to the ASM in asthma but do not affect ASM cell number, size or survival in co‐culture

**Figure 1:**
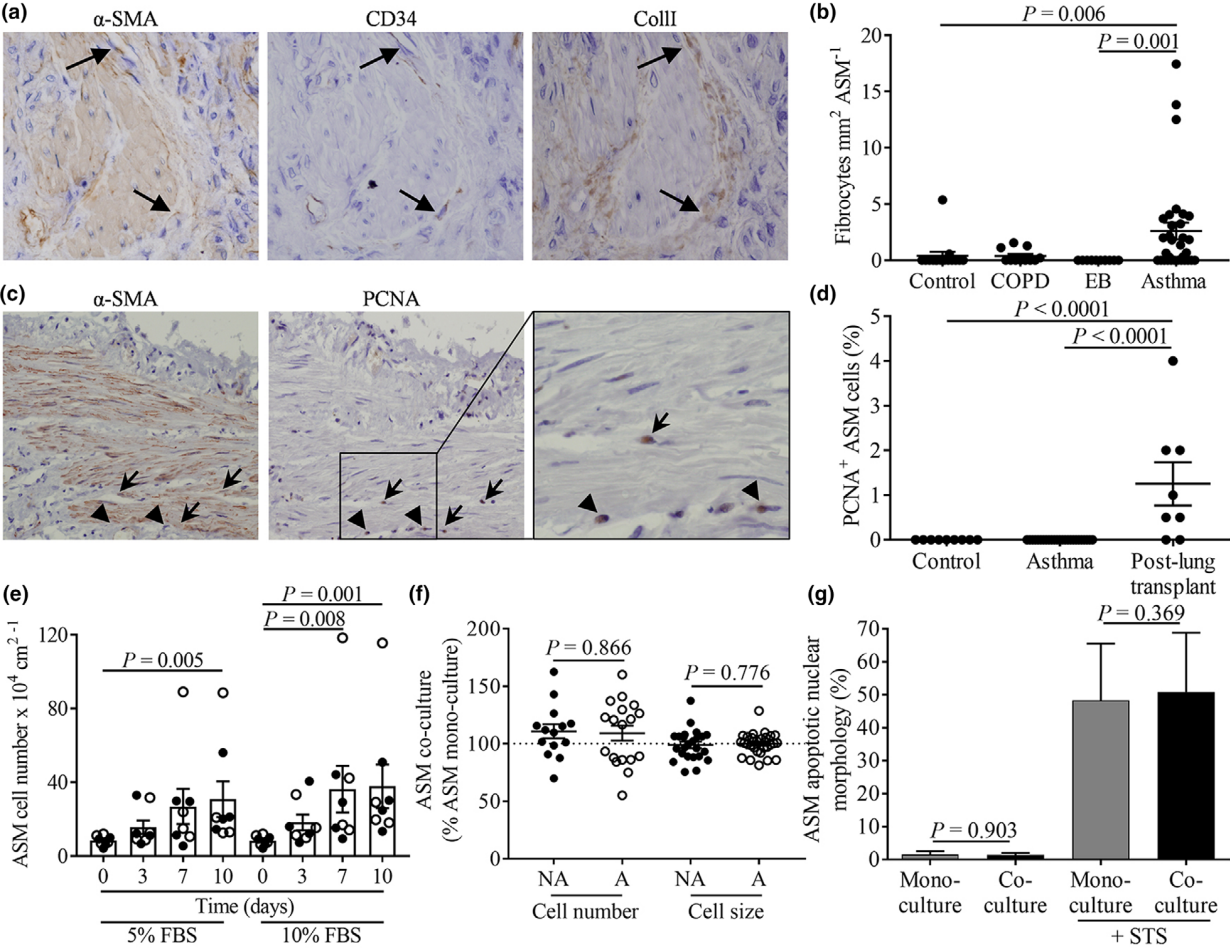
Fibrocytes localise to the ASM in asthma but do not affect ASM cell number, cell size or survival in co‐culture. **(a)** Representative photomicrographs showing α‐SMA, CD34 and CollI staining in sequential bronchial biopsy sections from an asthmatic patient. **(b)** Enumeration of α‐SMA, CD34 and CollI^+^ fibrocytes in the ASM in bronchial tissue sections from asthmatic (*n* = 32), COPD (*n* = 11) and NAEB (*n* = 12) patients versus controls (*n* = 15); *P* < 0.05, Kruskal–Wallis test, *P*‐values on figure from Dunn's multiple comparison test. **(c)** Representative photomicrographs showing α‐SMA and PCNA staining in sequential sections of positive control tissue from lung transplant recipients. **(d)** Enumeration of α‐SMA PCNA^+^ cells within the ASM in bronchial biopsy sections from controls (*n* = 9) versus asthmatic patients (*n* = 25), and in positive control tissue from lung transplant recipients (*n* = 8); *P* < 0.05, Kruskal–Wallis test, *P*‐values on figure from Dunn's multiple comparison test. **(e)** ASM cells from nonasthmatics (●, *n* = 4 ASM donors) and asthmatics (○, *n* = 4 ASM donors) were seeded at 10 × 10^4^ cells per cm^2^ of the well area and cell number in DMEM containing 5% or 10% FBS assessed over 10 days (Kruskal–Wallis; *P* < 0.05 for both 5% and 10% FBS, *P*‐values on figure for Dunn's multiple comparison test versus day 0). **(f)** ASM cell number following co‐culture of ASM cells with peripheral blood‐derived fibrocytes expressed as a % of respective ASM mono‐culture: ASM from nonasthmatics (NA), *n* = 14 different co‐culture experiments (with fibrocytes from 13 different donors co‐cultured with ASM from 7 different donors), *P* = 0.103 versus mono‐culture, paired *t*‐test; ASM from asthmatics (A), *n* = 18 different co‐culture experiments (with fibrocytes from 15 different donors co‐cultured with ASM from 11 different donors), *P* = 0.174 versus mono‐culture, paired *t*‐test; and ASM cell size following co‐culture of ASM cells with peripheral blood‐derived fibrocytes expressed as a % of respective ASM mono‐culture: ASM from NA, *n* = 24 different co‐culture experiments (with fibrocytes from 23 different donors co‐cultured with ASM from 11 different donors), *P* = 0.724 versus mono‐culture, paired *t*‐test; ASM from A, *n* = 30 different co‐culture experiments (with fibrocytes from 26 different donors co‐cultured with ASM from 18 different donors), *P* = 0.855 versus mono‐culture, Wilcoxon signed‐rank test. **(g)** Percentage of ASM population exhibiting apoptotic nuclear morphology, *n* = 4 different co‐culture experiments (with fibrocytes from 2 different donors co‐cultured with ASM from 3 different donors), following co‐culture with peripheral blood‐derived fibrocytes in the absence (*P* = 0.903 versus mono‐culture) and presence (*P* = 0.369 versus mono‐culture) of the positive control staurosporine (*P* < 0.05 versus untreated). Statistical analysis was performed using *t*‐tests. Data are presented as mean ± sem.

We present novel findings that fibrocyte number was significantly elevated in ASM in asthma versus NAEB and that fibrocytes are not significantly elevated in ASM in NAEB or COPD versus controls, and confirm previous findings that fibrocytes are elevated in ASM in asthma versus controls (fibrocytes per mm^2^ ASM, controls: 0.40 ± 0.36; COPD: 0.38 ± 0.19; NAEB: 0.0 ± 0.0; asthma: 2.61 ± 0.75, Figure 1a, b). For clinical characteristics, see Table 1. Fibrocyte number in the ASM correlated weakly with age of asthma onset (Supplementary figure 1a) and increased significantly in late‐onset versus early‐onset asthmatics (fibrocytes mm^2^ ASM: 3.7 ± 1.7 versus 1.4 ± 0.84, respectively, Supplementary figure 1b), whereas ASM mass was not significantly different between early‐onset versus late‐onset asthmatics (*P* = 0.472). Correlations performed with fibrocytes mm^2^ ASM against other clinical characteristics detailed in the Methods were not significant.

**Table 1:** Subject characteristics used for IHC of fibrocyte localisation to ASM

	Control (*n* = 15)	COPD (*n* = 11)	NAEB (*n* = 12)	Asthmatic (*n* = 32)	ANOVA/Kruskal–Wallis^$^/Chi‐squared test^
Age, years^a^	55 ± 3	64 ± 2	49 ± 4*	54 ± 2*	*P* = 0.017
Males *n* (%)	12 (80)	9 (82)	4 (33)	18 (56)	*P* = 0.037^
Smoking history, non, ever (*n*)	5, 10	0, 11	10, 2	32, 0	*P* < 0.001^
Pack years,^a^	21 ± 4	37 ± 6	5 ± 30.4*, ^#^	1 ± 0.4*, ^#^	*P* < 0.001^$^
ICS (μg day^−1^ BDP equivalent)^b^	0 (0–0)	0 (0–0)	0 (0–700)	1600 (1000–1600)*, ^#^, ^ʃ^	*P* < 0.001^$^
Pre‐BD FEV_1_ % predicted^b^	86 (14)	67 (22)^#^	97 (18)*	71 (50)^#^, ^ʃ^	*P* < 0.001^$^
Pre‐BD FEV_1_/FVC^b^	80 (10)	56 (17)^#^	85 (16)*	67 (24)^#^, ^ʃ^	*P* < 0.001
Source of tissue	Resection	Resection	Biopsy	Biopsy	

Clinical characteristics of subjects from which tissue for immunohistochemistry were derived.

ICS, inhaled corticosteroids; BDP, beclometasone dipropionate; FEV_1_, forced expiratory volume in 1 s; FVC, forced vital capacity; pre‐BD, prebronchodilator.

^a^ Mean ± SEM.

^b^ Median (interquartile range).

* *P* < 0.05 versus COPD,

^#^ *P* < 0.05 versus control,

^ʃ^ *P* < 0.05 versus NAEB.

*P*‐values without a symbol were derived using an ANOVA test. $ denotes use of a Kruskal–Wallis test. ^ denotes use of a Chi‐squared test.

PCNA^+^‐proliferating cells were not detected in ASM in asthma versus controls but could be detected in ASM in positive control tissue from lung transplant recipients (1.25 ± 0.48%, Figure 1c and d). Primary ASM cells from nonasthmatics versus asthmatics seeded at different densities (0.5–20 × 10^4^ ASM cells per cm^2^ of the well area in media containing 5 or 10% FBS) increased in number after 7–10 days of culture (Figure 1e, using 10 × 10^4^ ASM cells per cm^2^ of the well area as an exemplar); however, cell number was not different between ASM cells from nonasthmatics versus asthmatics at any seeding density or timepoint (*P* > 0.05, unpaired *t*‐test/Mann–Whitney *U*‐test as appropriate). To provide scope for proliferation to occur, ASM cells were seeded at a density of 7 × 10^4^ cells per cm^2^ of the well area and co‐cultured with peripheral blood‐derived fibrocytes. A small but insignificant increase in ASM cell number was observed following co‐culture compared to mono‐culture, as assessed by cell counts (cell number % ASM mono‐culture: ASM from nonasthmatics: 110.9 ± 6.2%; ASM from asthmatics: 109.3 ± 6.5%), with no difference in the change in cell number between ASM cells from asthmatics versus nonasthmatics (Figure 1f, *P* = 0.866, unpaired *t*‐test).

ASM cell size measured by geometric mean (GM) of the forward scatter arbitrary value (FSC) was not significantly different following co‐culture of ASM cells with peripheral blood‐derived fibrocytes compared to respective mono‐cultures (cell size % ASM mono‐culture: ASM from nonasthmatics: 99.0 ± 2.8%; ASM from asthmatics: 100.0 ± 1.7%, Figure 1f), with no difference in the change in cell size between ASM cells from asthmatics versus nonasthmatics (Figure 1f, *P* = 0.776, Mann–Whitney *U*‐test). In addition to there being no change in cell size, the percentage of ASM cells with apoptotic nuclear morphology was unaffected following co‐culture with fibrocytes either at baseline or post‐treatment with pro‐apoptotic staurosporine (STS, 0.1 µM, 20 h, mono‐culture: 1.6 ± 1.0% versus co‐culture: 1.4 ± 0.6%; mono‐culture + STS: 48.3 ± 17.2% versus co‐culture + STS: 50.8 ± 18.0%, Figure 1g).

### α‐SMA and TGFβR1 expression levels are greater in fibrocytes following co‐culture with ASM from asthmatics versus nonasthmatics and correlate with each other

**Figure 2:**
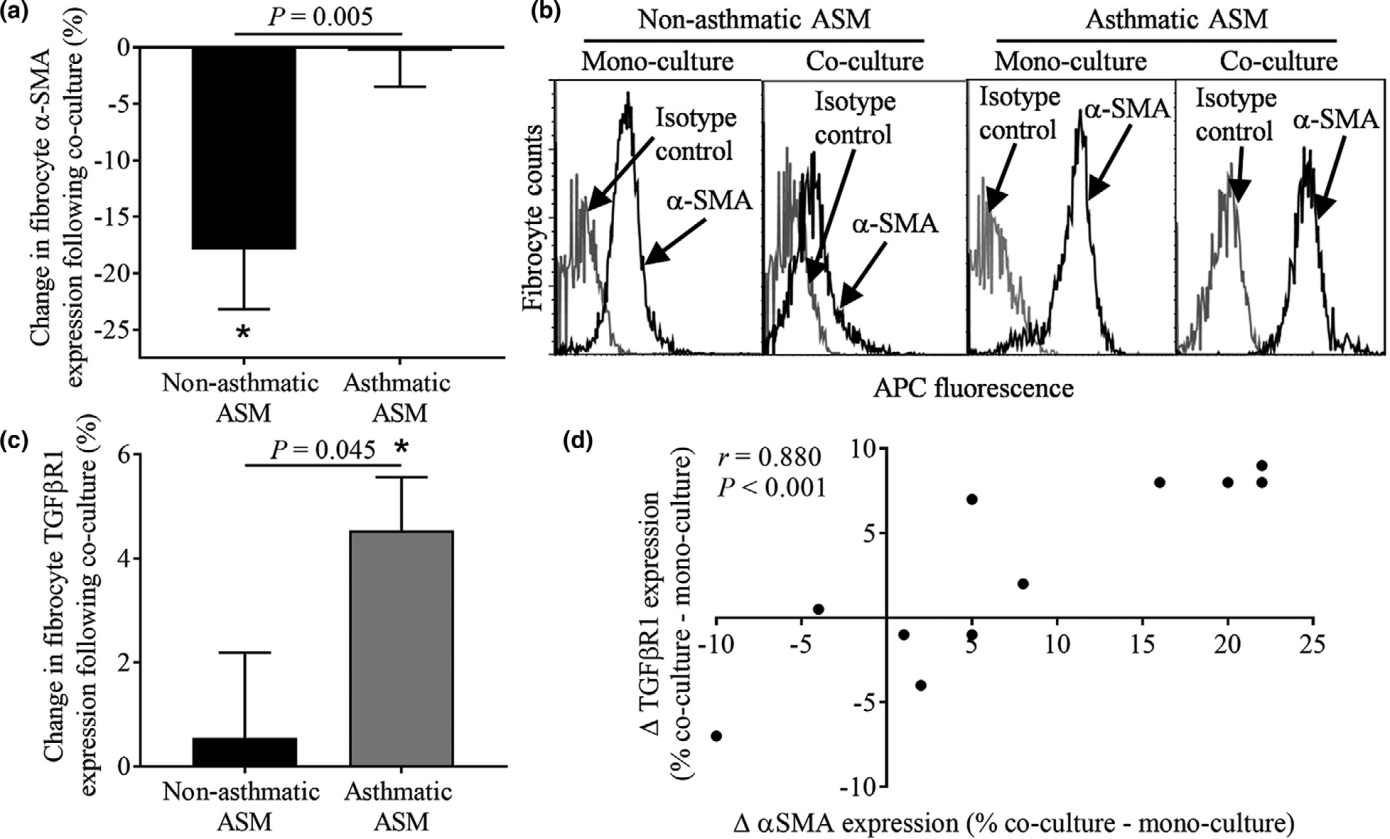
α‐SMA and TGFβR1 expression levels are greater in peripheral blood‐derived fibrocytes following co‐culture with ASM from asthmatics versus nonasthmatics and correlate with each other. **(a)** Change in percentage of fibrocytes expressing α‐SMA following co‐culture with ASM from nonasthmatics, *n* = 22 different co‐culture experiments (with fibrocytes from 20 different donors co‐cultured with ASM from 14 different donors), * *P* = 0.003 or asthmatics, *n* = 26 different co‐culture experiments (with fibrocytes from 23 different donors co‐cultured with ASM from 18 different donors), *P* = 0.929, versus mono‐culture (two‐tailed paired *t*‐tests). Data are expressed as mean ± sem. **(b)** Example flow cytometric histograms showing fibrocyte α‐SMA expression (black lines) versus isotype control (grey lines) in mono‐culture and co‐culture with ASM from nonasthmatics and asthmatics. **(c)** Change in percentage of fibrocytes expressing TGFβR1 following co‐culture with ASM from nonasthmatics, *n* = 10 different co‐culture experiments (with fibrocytes from 10 different donors co‐cultured with ASM from 6 different donors), *P* = 0.745, or asthmatics, *n* = 12 different co‐culture experiments (with fibrocytes from 10 different donors co‐cultured with ASM from 8 different donors), *P = 0.001 versus mono‐culture (two‐tailed paired *t*‐tests). Data are expressed as mean ± sem. **(a)** and **(c)**: *P*‐values on graphs represent comparison of expression levels (expressed as percentage change versus respective FC mono‐cultures) in fibrocytes following co‐culture with ASM from nonasthmatics versus asthmatics (two‐tailed unpaired *t*‐tests). **(d)** In a subset of donors, both fibrocyte α‐SMA and TGFβR1 expression levels were assessed, *n* = 11 different co‐culture experiments (with fibrocytes from 5 different donors co‐cultured with ASM from 5 different donors) with the change in expression levels between mono‐culture and ASM co‐culture shown to positively correlate (*r* = 0.88, *P* = 0.004, Pearson's correlation).

Peripheral blood‐derived fibrocytes expressed markers of smooth muscle differentiation in mono‐culture (α‐smooth muscle actin (α‐SMA): 80.9 ± 2.9%, *n* = 48; h‐caldesmon: 80.3 ± 7.0%, *n* = 14; myosin heavy chain (MHC): 11.9 ± 2.8%, *n* = 13), with expression maintained following co‐culture with ASM from asthmatics and reduced following co‐culture with ASM from nonasthmatics compared to respective mono‐cultures (Figure 2a and b (α‐SMA), Supplementary figure 2a and b (h‐caldesmon and MHC, respectively) and Table 2). TGF‐β1 levels in supernatants (SNs) were unaffected by fibrocyte/ASM co‐culture (Supplementary figure 3a); however, fibrocyte TGF‐β1 type 1 receptor (TGFβR1) expression increased following co‐culture with ASM from asthmatics but not nonasthmatics (Figure 2c, Table 2). Interestingly, where both were measured, changes in TGFβR1 and α‐SMA expression were correlated (*r* = 0.88, *P* < 0.001, Figure 2d).

**Table 2:** α‐SMA, h‐caldesmon, MHC and TGFβR1 expression levels in peripheral blood‐derived fibrocytes following co‐culture with ASM from nonasthmatics and asthmatics

	Expression in fibrocytes following co‐culture with nonasthmatic ASM (% population)				Expression in fibrocytes following co‐culture with asthmatic ASM (% population)			
	Mono‐culture	Co‐culture	% change	*P*‐value, *n* number	Mono‐culture	Co‐culture	% change	*P*‐value, *n* number
α‐SMA	76.2 ± 5.0	58.4 ± 7.2	−17.9 ± 5.3	*P* = 0.003, *n* = 22 (20,14)	84.7 ± 3.1	84.4 ± 1.9	−0.3 ± 3.2	*P* = 0.928, *n* = 26 (23,18)
h‐Caldesmon	84.3 ± 4.9	62.5 ± 11.4	−21.8 ± 8.1	*P* = 0.043, *n* = 6 (5,4)	77.25 ± 11.9	76.6 ± 8.9	−0.63 ± 9.7	*P* = 0.950, *n* = 8 (7,5)
MHC	13.8 ± 4.8	4.9 ± 1.4	−8.9 ± 3.7	*P* = 0.054, *n* = 7 (7,5)	9.6 ± 2.7	6.5 ± 2.2	−3.1 ± 1.6	*P* = 0.105, *n* = 6 (5,5)
TGFβR1	92.2 ± 1.4	92.7 ± 1.8	0.55 ± 1.6	*P* = 0.745, *n* = 10 (10,6)	90.1 ± 1.4	94.7 ± 1.3	4.5 ± 1.0	*P* = 0.001, *n* = 12 (11,9)

Peripheral blood‐derived fibrocytes were co‐cultured with ASM isolated from nonasthmatic or asthmatic donors for 7–8 days. α‐SMA, h‐caldesmon, MHC and TGFβR1 expression levels were assessed by flow cytometry in CFSE‐labelled fibrocytes following co‐culture and in parallel Mono‐cultures and expressed as % population versus respective isotype controls and additionally as percentage change in co‐cultures versus respective fibrocyte mono‐cultures. Data are expressed as mean ± SEM. *P*‐values from paired *t*‐tests of fibrocyte α‐SMA, h‐caldesmon, MHC and TGFβR1 expression levels in co‐culture versus respective mono‐cultures.

In contrast, co‐culture of ASM cells with peripheral blood‐derived fibrocytes had no effect on ASM α‐SMA, h‐caldesmon or MHC expression (Supplementary figure 3b–d) or TGFβR1 expression in ASM from asthmatics, but resulted in a small reduction in TGFβR1 expression in ASM from nonasthmatics (Supplementary figure 3e). Following fibrocyte/ASM co‐culture, intercellular adhesion molecule‐1 (ICAM‐1) expression was increased, but not significantly, in fibrocytes (mono‐culture: 80.3 ± 6.3% versus co‐culture: 97.7 ± 0.3%) and increased significantly in ASM (mono‐culture: 85.0 ± 0.6% versus co‐culture: 97 ± 0.9%, Supplementary figure 3f).

### Fibrocyte/ASM co‐culture results in enhanced contraction of collagen gels

**Figure 3:**
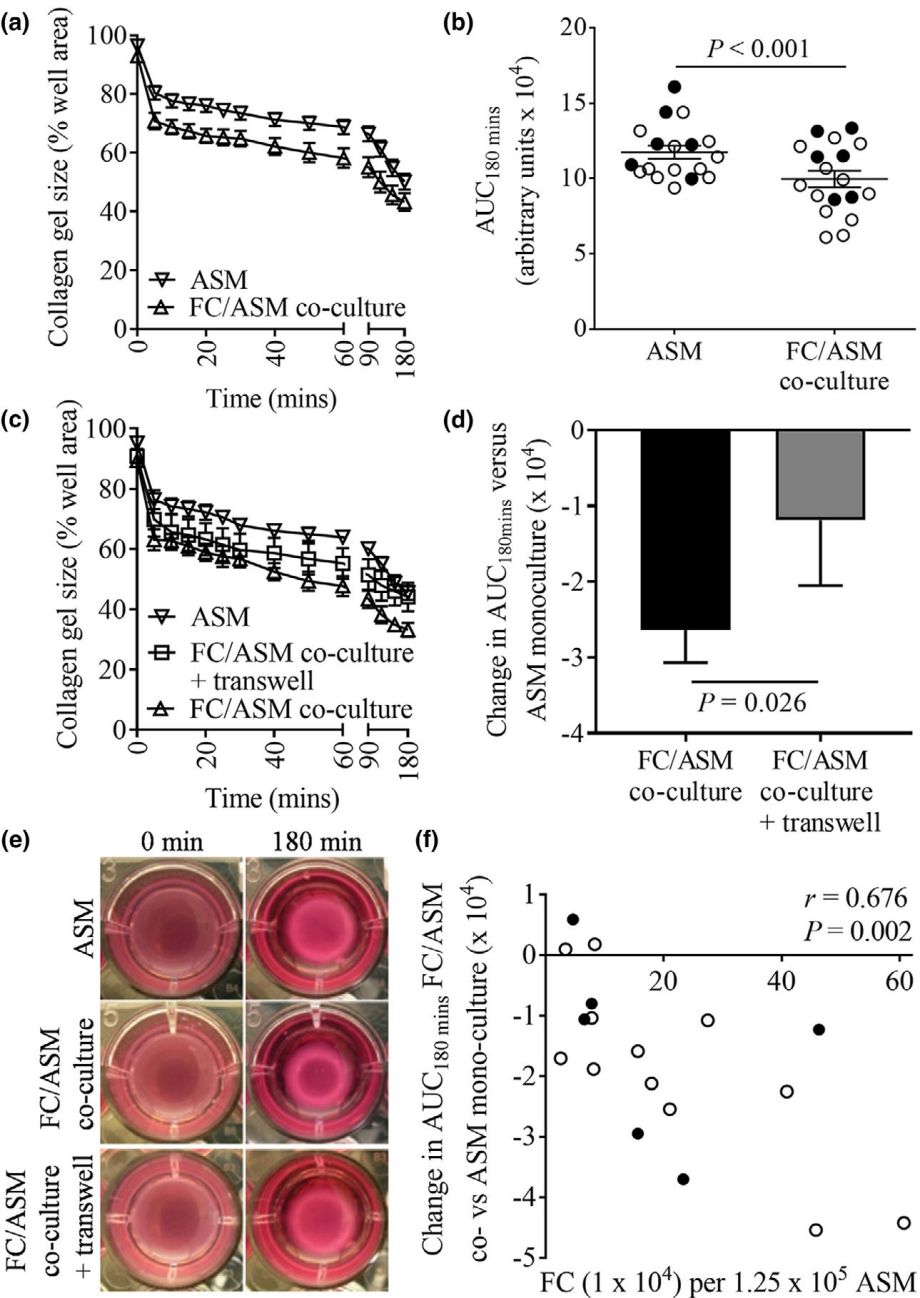
Co‐cultures of ASM cells with peripheral blood‐derived fibrocytes results in enhanced bradykinin‐induced contraction of collagen gels. **(a)** Time course and **(b)** area under curve over 180 min (AUC_180min_) of bradykinin‐stimulated contraction of collagen gels impregnated with fibrocytes (FC)/ASM co‐cultured for 3–4 days versus ASM alone (AUC_180min_ all experiments: *n* = 18 different co‐culture experiments (with fibrocytes from 16 different donors co‐cultured with ASM from 11 different donors), *P* < 0.001; ASM from nonasthmatics (●):*n* = 6 different co‐culture experiments (with fibrocytes from 5 different donors co‐cultured with ASM from 5 different donors), *P* = 0.06; ASM from asthmatics (○):*n* = 12 different co‐culture experiments (with fibrocytes from 11 different donors co‐cultured with ASM from 6 different donors), *P* < 0.001). **(c)** Time course and **(d)** change in AUC_180min_ of bradykinin‐stimulated contraction of collagen gels impregnated with fibrocytes/ASM co‐cultured for 3–4 days with direct cell–cell contact (FC/ASM co‐culture) or with fibrocytes seeded onto Transwell inserts (FC/ASM co‐culture + Transwell) versus ASM mono‐culture, *n* = 9 different co‐culture experiments (with fibrocytes from 9 different donors co‐cultured with ASM from 6 different donors), *P* = 0.026, paired *t*‐test. Data are plotted as mean ± sem. **(e)** Example photomicrographs of collagen gels impregnated with ASM, FC/ASM co‐cultures and FC/ASM co‐culture + Transwells at 0 and 180 min. **(f)** Correlation of the change in AUC_180min_ between collagen gels impregnated with ASM mono‐cultures and fibrocyte/ASM co‐cultures with the number of fibrocytes present per 1.25 × 10^5^ ASM, all experiments: *n* = 18 different co‐culture experiments (with fibrocytes from 16 different donors co‐cultured with ASM from 11 different donors), *r* = 0.676, *P* = 0.002; ASM from nonasthmatics (●):*n* = 6 different co‐culture experiments (with fibrocytes from 5 different donors co‐cultured with ASM from 5 different donors), *r* = 0.332, *P* = 0.520; and ASM from asthmatics (○):*n* = 12 different co‐culture experiments (with fibrocytes from 11 different donors co‐cultured with ASM from 6 different donors), *r* = 0.813, *P* = 0.001, Pearson's correlations.

Following co‐culture of ASM cells with peripheral blood‐derived fibrocytes, bradykinin‐induced collagen gel contraction increased versus ASM alone, as indicated by a reduction in collagen gel size and a decrease in the area under the curve (AUC) over 180 min (AUC_180min_ co‐culture: 9960 ± 545 versus ASM: 11738 ± 1830, Figure 3a and b). This was partially dependent on changes within the ASM cells and/or fibrocytes or mediators released at a higher local concentration following sustained cell–cell contact, as the enhanced bradykinin‐induced contraction was inhibited when fibrocyte–ASM adhesion was blocked by Transwell inserts (reduction in AUC_180min_ versus ASM mono‐culture: co‐culture, 2638 ± 431; co‐culture plus Transwell, 1189 ± 863, Figure 3c–e), and was not observed when ASM contraction was stimulated in the presence of co‐culture SNs (Supplementary figure 4a–c). To confirm whether sustained cell–cell contact was required to observe the enhanced contraction seen following fibrocyte/ASM co‐culture, collagen gels were impregnated with fibrocytes and ASM cells cultured separately prior to experimentation at the range of ratios observed following co‐culture. No increase in bradykinin‐induced contraction was observed (Supplementary figure 4d–f), confirming the requirement for sustained cell–cell contact. Additionally, in a subset of experiments, fibrocytes were added alone to collagen gels at a density of 31.25 × 10^4^ cells per gel, equivalent to that found in a 1:4 ratio with ASM, with no bradykinin‐induced contraction observed (collagen gel size (% well area) 0 min; 99 ± 4 versus 180 min; 97 ± 1, *n* = 5, *P* = 0.17, paired *t*‐test).

Interestingly, the change in contraction between fibrocyte/ASM co‐culture and ASM alone positively correlated with the number of fibrocytes present per collagen gel, that is the number of fibrocytes present per 1.25 × 10^5^ ASM cells following co‐culture (*r* = 0.676, *P* = 0.002, Figure 3f). This correlation and the increase in contraction remained significant when considering data from fibrocytes co‐cultured with ASM from asthmatics but not nonasthmatics (Figure 3b and f).

### Fibrocytes demonstrate increased granularity and plasticity following ASM co‐culture which is dependent on the inflammatory milieu

**Figure 4:**
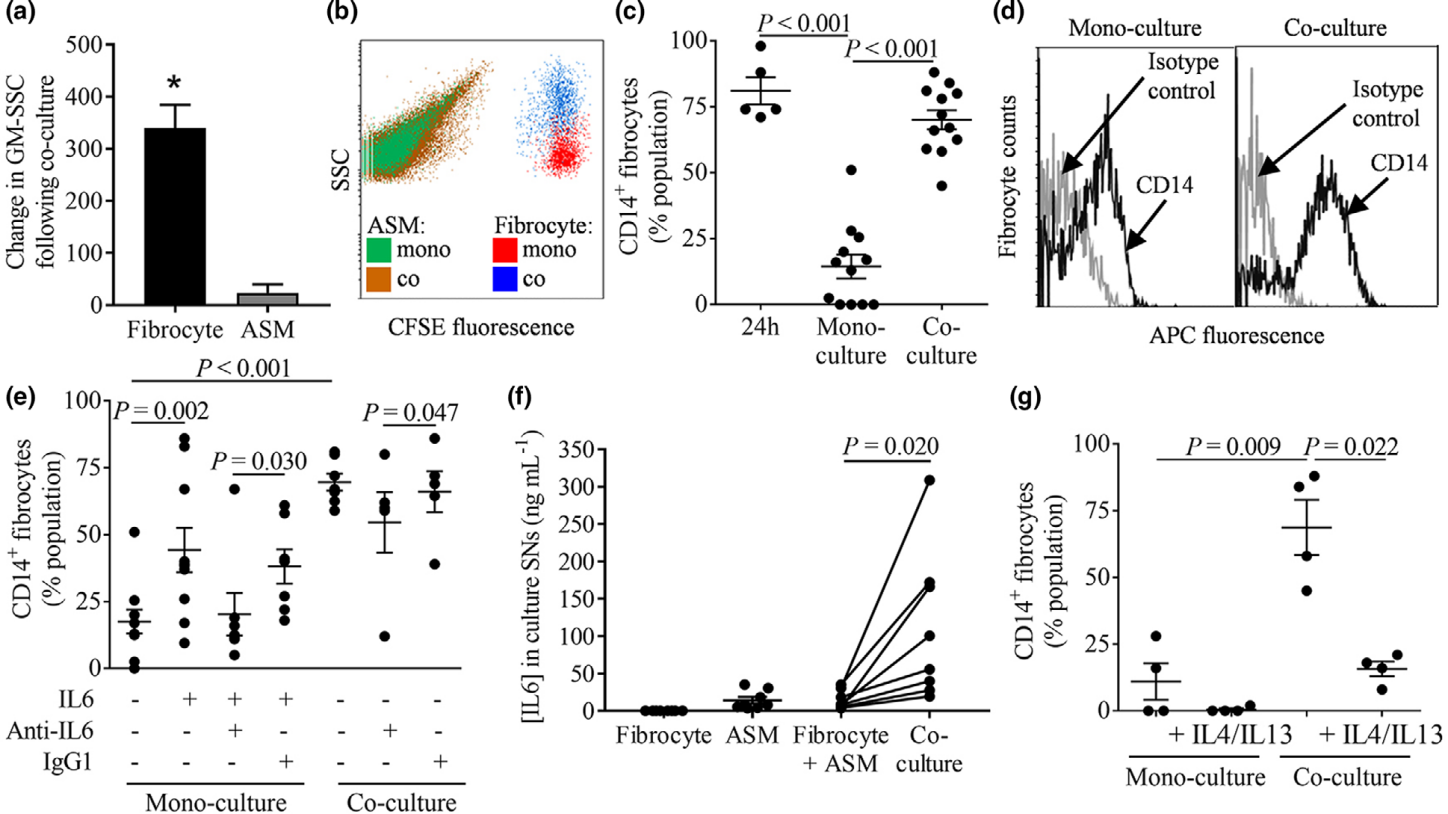
Peripheral blood‐derived fibrocytes demonstrate increased granularity and plasticity following co‐culture with ASM which is dependent on the inflammatory milieu. **(a)** Change in granularity (SSC) of fibrocytes, *n* = 66 different co‐culture experiments (with fibrocytes from 45 different donors co‐cultured with ASM from 32 different donors), and ASM, *n* = 54 different co‐culture experiments (with fibrocytes from 34 different donors co‐cultured with ASM from 29 different donors) following fibrocyte/ASM co‐cultures compared to respective mono‐cultures. * denotes *P* < 0.05 versus mono‐culture. **(b)** Example flow cytometric dot plots of SSC plotted against CFSE fluorescence for unlabelled ASM and CFSE‐labelled fibrocytes in mono‐culture (mono) versus co‐culture (co). **(c)** CD14 expression in fibrocytes following 7 days of mono‐culture is reduced compared to the PBMC population adhered to FN‐coated TC flasks 24h after isolation (*n* = 5 fibrocyte donors, *P* < 0.001, unpaired *t*‐test) but is restored following co‐culture with ASM cells, *n* = 12 different co‐culture experiments (with fibrocytes from 12 different donors co‐cultured with ASM from 10 different donors), *P* < 0.001 versus mono‐culture, paired *t*‐test. **(d)** Example flow cytometric histograms showing fibrocyte CD14 expression (black lines) versus isotype control (grey lines) in mono‐culture and following co‐culture with ASM. **(e)**  In mono‐culture, fibrocyte CD14 expression is increased following incubation with 100 ng mL^−1^ IL‐6 for 7‐8 days, *n* = 10 fibrocyte donors, *P* = 0.002, paired *t*‐test. IL‐6‐induced fibrocyte CD14 expression is reduced following incubation with 10 µg mL^−1^ anti‐IL‐6 compared to IgG1 isotype control, *n* = 7 fibrocyte donors, *P* = 0.030, paired *t*‐test. Fibrocyte CD14 expression following ASM co‐culture is reduced following anti‐IL‐6 blockade of endogenous IL‐6 versus IgG1 isotype control, *n* = 5 different co‐culture experiments (with fibrocytes from 5 different donors co‐cultured with ASM from 5 different donors), *P* = 0.047, paired *t*‐test. **(f)** IL‐6 (ng mL^−1^) present in culture SNs from fibrocytes co‐cultured with ASM is increased versus the sum of IL‐6 (ng mL^−1^) in corresponding fibrocyte/ASM mono‐cultures, *n* = 8 different co‐culture experiments (with fibrocytes from 7 different donors co‐cultured with ASM from 6 different donors), *P* = 0.020, paired *t*‐test. **(g)** Effect of incubation with 10 ng mL^−1^ IL‐4/IL‐13 for 7–8 days on fibrocyte CD14 expression following mono‐culture and co‐culture with ASM, *n* = 4 different co‐culture experiments (with fibrocytes from 4 different donors mono‐cultured or co‐cultured with ASM from 4 different donors), *P* = 0.022, paired *t*‐test. Data are presented as mean ± sem; statistical analysis was performed using *t*‐tests.

Following co‐culture of peripheral blood‐derived fibrocytes with ASM cells, fibrocyte granularity significantly increased (mean difference GM‐side scatter arbitrary value (SSC): 339 ± 45, Figure 4a and b) with no difference between co‐culture with ASM from nonasthmatics (*n* = 28 different co‐culture experiments (with fibrocytes from 27 different donors co‐cultured with ASM from 12 different donors)) versus asthmatics (*n* = 38 different co‐culture experiments (with fibrocytes from 33 different donors co‐cultured with ASM from 20 different donors), *P* = 0.686, unpaired *t*‐test). In contrast, ASM granularity was unaffected (mean difference SSC; 23 ± 17, *P* = 0.164, Wilcoxon signed‐rank test, Figure 4a and b), as was fibrocyte size (mean difference GM‐FSC, −109.6 ± 76.9, *n* = 66 different co‐culture experiments (with fibrocytes from 45 different donors and ASM from 32 different donors), *P* = 0.378, Wilcoxon signed‐rank test).

Fibrocytes derive from a highly CD14^+^ peripheral blood cell population (CD14^+^ adherent cells 24h post isolation from peripheral blood: 81.0 ± 5.0%, *n* = 5, Figure 4c). After differentiation into fibrocytes over 5–10 days, CD14 expression is reduced (23.8 ± 6.1%) and remained low following 7 days in mono‐culture but was restored in parallel fibrocyte/ASM co‐cultures (14.4 ± 4.5% versus 70.0 ± 3.67%, Figure 4c and d), with no difference seen between co‐culture with ASM from nonasthmatics (*n* = 6 different co‐culture experiments (with fibrocytes from 6 different donors co‐cultured with ASM from 5 different donors)) versus asthmatics (*n* = 6 different co‐culture experiments (with fibrocytes from 6 different donors co‐cultured with ASM from 5 different donors), *P* = 0.693, unpaired *t*‐test). In a subset of fibrocyte donors exhibiting CD14 expression (% CD14‐positive population: 75.0 ± 6.3, *n* = 4 different co‐culture experiments (with fibrocytes from 4 different donors co‐cultured with ASM from 4 different donors)), α‐SMA expression after co‐culture with ASM cells was also measured (% positive α‐SMA population: 77.9 ± 6.8 (*n* = 4 different co‐culture experiments (with fibrocytes from 4 different donors co‐cultured with ASM from 4 different donors)).

Peripheral blood‐derived fibrocytes were incubated with mediators expressed by fibrocytes/ASM alone or following co‐culture (CCL11, CCL19, CXCL10, CX3CL1, IL‐6, IL‐8, IL‐1β, TNF‐α, SDF1α and CCL2). Of these, only IL‐6 significantly increased fibrocyte CD14 expression, confirmed by blockade with an IL‐6‐neutralising antibody (anti‐IL‐6) (% CD14^+^ fibrocytes; control: 17.6 ± 4.4%; 100 ng mL^−1^ IL‐6: 44.3 ± 8.3%; 100 ng mL^−1^ IL‐6 + isotype control: 38.1 ± 6.4%; 100 ng mL^−1^ IL‐6 + anti‐IL‐6: 20.3 ± 8.0%, Figure 4e). In addition, blockade of endogenous IL‐6 with an IL‐6‐neutralising antibody significantly inhibited fibrocyte CD14 expression compared to an isotype control antibody following fibrocyte/ASM co‐culture (% CD14^+^ fibrocytes; co‐culture + isotype control: 66.1 ± 7.7%; co‐culture + anti‐IL‐6: 54.6 ± 11.3%; Figure 4e), and IL‐6 levels in fibrocyte/ASM co‐culture SNs were increased compared to the sum of respective mono‐cultures (Ʃ fibrocyte/ASM: 14.2 ± 4.4 pg mL^−1^; co‐culture: 111.2 ± 35.2 pg mL^−1^, Figure 4f). This was in part because of a co‐operative response between fibrocytes and ASM, as the amount of IL‐6 in SNs following co‐culture was significantly reduced when cell–cell adhesion was blocked with a Transwell insert (% reduction: 61.9 ± 9.4, *n* = 4 different co‐culture experiments (with fibrocytes from 4 different donors co‐cultured with ASM from 4 different donors), *P* = 0.027). In contrast to IL‐6, the T2 cytokines IL‐4/IL‐13 inhibited the increased fibrocyte CD14 expression following fibrocyte/ASM co‐culture (co‐culture: 68.8 ± 10.3%; co‐culture with IL‐4/IL‐13: 15.8 ± 2.8%, Figure 4g).

## Discussion

We show for the first time that fibrocyte numbers in the ASM in asthma are increased compared to NAEB and that fibrocyte numbers are not significantly elevated in the ASM in NAEB or COPD versus controls, and confirm previous findings that fibrocyte numbers are elevated in the ASM in asthma versus controls. We show no detectable difference in ASM proliferation in asthmatics versus healthy controls *in vivo* and no difference in ASM proliferation, apoptotic status or size following culture with or without peripheral blood‐derived fibrocytes. Following fibrocyte co‐culture with ASM from asthmatics, compared to nonasthmatics, both fibrocyte smooth muscle marker expression and collagen gel contraction are greater. Thus, recruitment of fibrocytes to the ASM bundle in asthma has the potential to contribute to increased ASM mass and ASM hypercontractility.

Increased ASM mass in asthma is related to lung function decline, worse asthma control and more exacerbations.^11^, ^12^ However, to date, only two investigational medicinal products, fevipiprant and gallopamil, and one nonpharmacological intervention, bronchial thermoplasty, have shown any potential in reducing ASM mass in clinical trials, with the reduction in ASM mass following fevipiprant treatment correlating with reduced fibrocyte number in the lamina propria, supporting a role for precursor cell recruitment in increased ASM mass.^10^, ^13^, ^14^ Subsequent phase 3 trials with fevipiprant have not demonstrated significant improvement in lung function;^15^, ^16^ however, patients were not stratified according to their ASM mass. Thus, whether fibrocyte localisation to ASM results in meaningful clinical outcomes requires further understanding of how fibrocytes affect ASM phenotype and behaviour, which could help identification of appropriate primary outcomes to measure in the future.

The increased ASM mass in asthma has been shown to be a result of both ASM cell hypertrophy and hyperplasia,^17^ with enhanced ASM proliferation, prolonged ASM survival or migration of ASM cells/precursors to the ASM bundle proposed to contribute to hyperplasia and asthma‐relevant mediators shown to induce ASM hypertrophy.^8^, ^18^ However, a consensus on the underlying mechanism is lacking. We are unable to detect PCNA expression, a marker of proliferation, in ASM bundles from asthmatics *in vivo*. This is supported *in vitro* with no differences in proliferation of ASM from asthmatics versus nonasthmatics observed *per se*. In addition, a small but insignificant increase in the number of ASM cells from both nonasthmatics and asthmatics following co‐culture with peripheral blood‐derived fibrocytes was observed, with no difference between the two groups, supporting the observations of Lin *et al*.^19^, in which Ki67 is used as a marker of proliferation. Moreover, we see no evidence of hypertrophy or reduced apoptosis in the ASM *per se* and/or following co‐culture with peripheral blood‐derived fibrocytes.

Taking these observations into account, we addressed whether fibrocytes have the potential to contribute to increased ASM mass *per se* by acquiring a smooth muscle‐like phenotype in asthma. We showed that peripheral blood‐derived fibrocytes expressed well‐recognised smooth muscle differentiation markers to a greater extent following direct cell–cell contact in co‐culture with ASM from asthmatics compared to nonasthmatics, whereas that of ASM cells was unchanged, in support of observations with ASM cells following Transwell culture with fibrocytes.^19^ TGF‐β1 is a key driver of fibrocyte differentiation and is implicated in airway remodelling in asthma.^20^, ^21^ TGF‐β1 levels were unchanged following fibrocyte/ASM co‐culture versus mono‐culture, as has been previously reported;^19^ however, fibrocyte TGFβR1 expression was increased following co‐culture with ASM from asthmatics, with the extent of change of TGFβR1 expression correlating with that of α‐SMA expression. Thus, fibrocytes may be more responsive to TGFβ1 following ASM co‐culture, with respect to phenotypic changes or contractile ability.

Next, we addressed whether fibrocyte/ASM interactions had an effect on the extent of contraction of collagen gels in response to an asthma‐relevant bronchoconstricting stimulus, bradykinin.^22^ We demonstrated that co‐culture of ASM cells with peripheral blood‐derived fibrocytes resulted in increased bradykinin‐induced collagen gel contraction versus ASM alone, which was greater following co‐culture with ASM from asthmatics versus nonasthmatics and correlated with fibrocyte number per 1.25 × 10^5^ ASM post co‐culture. This was partially dependent on changes within the ASM cells and/or fibrocytes *per se* or mediators released at a higher local concentration following sustained cell–cell contact. Interestingly, IL‐6, which is shown to be increased following fibrocyte/ASM co‐culture both in this study and by Lin *et al*. in 2014,^19^ can prime smooth muscle contraction in rat models and enhance ASM cell contraction in the presence of mast cells that are localised to ASM in asthma and implicated in AHR.^23^, ^24^, ^25^, ^26^ Additionally, fibrocyte/ASM co‐culture results in increased ICAM‐1 expression by ASM, which is linked to increased actomyosin contractility in fibroblasts.^27^ This study gives us a valuable insight into the effects of fibrocyte/ASM interactions following co‐culture in an *in vitro* model of contraction. However, one has to consider that although the ASM is a key driver of AHR, AHR can also be influenced by a number of additional factors *in vivo*, such as the inflammatory milieu, airway wall stiffness and airway‐parenchymal interactions.^28^ Unfortunately, airway hyper‐responsiveness was not assessed systematically in patients from which samples were taken. Further work is required to define the exact mechanism of increased collagen gel contraction following fibrocyte/ASM co‐culture and how this relates to AHR *in vivo*.

Our *in vitro* data and observations that fibrocyte numbers are increased in the ASM in asthma but not COPD, in which increased ASM mass is largely because of increased matrix deposition,^29^ support data from previous studies suggesting ASM/progenitor cell recruitment is a key contributor to increased ASM mass in asthma, with ASM‐derived mediators including PDGF implicated in this recruitment.^9^, ^10^, ^30^ Interestingly, type III receptor protein–tyrosine kinase inhibitors have shown promise in clinical trials in asthma.^31^ In addition, our observations that fibrocyte numbers are not increased in the ASM in NAEB which lacks the variable airflow obstruction and AHR seen in asthma support our data, suggesting that fibrocyte localisation to the ASM in asthma could contribute to the ASM hypercontractility and AHR seen in asthma. Indeed, asthma‐relevant mediators can stimulate fibrocyte α‐SMA expression and/or contraction of collagen gels,^20^, ^32^, ^33^ and fibrocytes from severe asthmatics have a greater differentiation potential.^34^, ^35^

The granularity of the peripheral blood‐derived fibrocytes increases following ASM co‐culture, indicative of increased cellular complexity. The functional consequence of this is unclear but could be related to protein secretion, activation status and collagen content, or endocytosis associated with antigen presentation.^36^, ^37^, ^38^ Consistent with this increased complexity, following fibrocyte/ASM co‐culture, fibrocytes regained the CD14 expression characteristic of the monocytic population from which they derive, in addition to maintaining α‐SMA expression following co‐culture with ASM from asthmatics. Such plasticity has been observed in other cells: CD14^+^ monocyte‐derived mesenchymal progenitors show morphologic and molecular features of monocytes as well as endothelial and mesenchymal cells,^39^ and mesodermal cells coexpress myofibroblast and macrophage markers to contribute to both membrane integrity and innate immunity^40^ enabling fulfilment of different biological needs. The partial IL‐6 dependence of CD14 expression, coupled with increased IL‐6 production following fibrocyte/ASM co‐culture, suggests a potential positive feedback loop, whereby fibrocyte/ASM co‐culture leads to increased IL‐6 production which upregulates CD14 expression^41^, ^42^ to further amplify IL‐6 production, as observed in several cell types.^43^, ^44^ Thus, following recruitment to the ASM, fibrocytes may retain the ability to contribute to monocyte‐mediated processes, as well as increasing levels of IL‐6, via interactions with ASM, both of which are implicated in asthma pathogenesis.^45^, ^46^

Interestingly, increased peripheral blood‐derived fibrocyte CD14 expression following co‐culture is abrogated by IL‐4/IL‐13, which are expressed by mast cells present in the ASM in asthma.^47^, ^48^ Thus, fibrocyte phenotype and consequent effects on ASM dysfunction in asthma could be influenced by the local inflammatory environment. In support of this Th‐1, Th‐2 and Th‐17 cytokines can differentially affect fibrocyte differentiation and phenotype.^49^, ^50^

Although the fibrocyte numbers within the ASM are lower *per se*, a similar fold increase in mast cell numbers within the ASM in asthma versus health, that is fourfold, versus the 6.5‐fold increase in fibrocyte numbers reported here, did relate to disease‐relevant outcomes.^26^ We interrogated fibrocyte number per mm^2^ ASM against available asthma patients' clinical characteristics, such as age, lung function and symptom scores (see Methods), to try to identify patient phenotypes that may be more susceptible to increased numbers of fibrocytes in their ASM. However, only a weak correlation with age of asthma onset was observed. Subsequent analysis of late‐onset versus early‐onset data suggested that fibrocyte localisation to the ASM may be slightly more predominant in late‐onset versus early‐onset asthma. As this was independent of ASM mass, this could suggest different mechanisms for increased ASM mass in these asthma phenotypes, for example increased proliferation could be more important in early‐onset asthma and inflammation, and ASM/progenitor cell recruitment could be more important in late‐onset asthma, or perhaps the role of fibrocytes in ASM dysfunction could be less prominent with disease duration. However, samples were obtained from patients with stable disease at a single timepoint in a cross‐sectional study; thus, we cannot exclude the possibility that fibrocyte numbers change over time or increase acutely during exacerbations to influence ASM function.

Bronchoscopy is an invasive procedure not suitable for most COPD patients, and availability of bronchoscopic tissue from control subjects is limited. Thus, assessment of fibrocyte number per mm^2^ ASM was performed using control and COPD samples from lung resection tissue derived from surgery, whereas NAEB and asthma tissue are from patients undergoing bronchoscopies as part of their clinical assessment. Consequently, the tissue may not be derived from comparable airways; however, our previous study with healthy tissue derived from bronchoscopies^9^ showed equivalent results to this study. In addition, two thirds of the control subjects are smokers; however, there is no difference in fibrocyte number per mm^2^ ASM between control smokers and nonsmokers in this study, controls in this and the previous study who were all nonsmokers,^9^ and no correlation across all subjects between fibrocyte number per mm^2^ ASM and pack‐year history. The COPD and control groups have a predominance of males and increased age; however, across all subjects, there is no difference in fibrocyte number fibrocyte number per mm^2^ ASM between males (60%) and females (40%) and no correlation between age and fibrocyte number per mm^2^ ASM. Another criticism could be that this study predominantly reflects fibrocyte localisation in eosinophilic asthmatics as the asthma patients have a mean sputum eosinophil percentage of 10 ± 2%; however, there is no difference in fibrocyte number per mm^2^ ASM between noneosinophilic and eosinophilic asthma in our previous study^9^, and no correlation between fibrocyte number per mm^2^ ASM and sputum eosinophil number in this study. In addition, one could argue that the increase in fibrocyte numbers in the ASM in asthma is driven by a subpopulation of 3 patients; however, we have not been able to detect any significant differences in the clinical characteristics of these patients compared to the rest of the asthma patients, and fibrocyte numbers in the ASM in asthma are still significantly increased compared to control and NAEB if these patients are excluded.

Peripheral blood‐derived fibrocytes and ASM have overlapping flow cytometric light scattering characteristics; thus, CFSE use has been instrumental in studying cell‐type‐specific changes in protein expression following fibrocyte/ASM co‐culture by enabling independent gating of fibrocyte and ASM populations for analysis. However, one of the main challenges of the study has been to demonstrate the contribution of cell‐type‐specific changes in fibrocytes and ASM to collagen gel contraction and therefore which cell type is likely to have the most impact clinically. The number of fibrocytes obtained from each donor is limited, and recovery of fibrocytes following adhesion to Transwells is poor meaning it was not possible to perform collagen gel contraction assays with fibrocytes *per se* or following cell sorting. However, by combining co‐culture experiments with experiments using culture SNs, fibrocytes and ASM cells with no prior co‐culture and Transwells to block fibrocyte/ASM adhesion, we have shown that enhanced collagen gel contraction is dependent on prior co‐culture of fibrocyte/ASM because of cell–cell adhesion and/or localised release of inflammatory mediators. The requirement for primary ASM from healthy and asthmatic donors at the same time as having sufficient cells from, and availability of, primary fibrocyte preparations from healthy and asthmatic donors meant we were unable to make comparisons in a checkerboard manner, which would have strengthened our observations. In addition, limitations in the number of fibrocytes isolated from some donors precluded the panel of smooth muscle markers being assessed in parallel in all donors and the use of multiple ASM:FC ratios; thus, a ratio of 2 ASM: 1 fibrocyte was chosen as this was shown to be the optimum ratio for detecting changes following co‐culture in another study.^19^

In summary, we show that fibrocyte recruitment to the ASM is a feature of asthma but not COPD or NAEB. Our *in vitro* data support a potential role for fibrocytes in increased ASM mass and ASM hypercontractility observed in asthma and suggest that fibrocytes may retain the ability to contribute to asthma pathogenesis via monocyte‐mediated processes, dependent on the composition of the local inflammatory milieu. Further understanding of the mechanisms of fibrocyte recruitment to and/or differentiation within the ASM may identify novel therapeutic targets to modulate ASM dysfunction in asthma.

## METHODS

### Subjects

Bronchial tissue for assessing fibrocyte localisation to the ASM was obtained from lung resection specimens from control subjects with normal lung function (*n* = 15) and patients with COPD (*n* = 11) defined using Global Initiative for Chronic Obstructive Lung Disease (GOLD) guidelines,^51^ and bronchial biopsies, obtained via video‐assisted bronchoscopy, from patients with nonasthmatic eosinophilic bronchitis (*n* = 12) defined using the American College of Chest Physicians guidelines^52^ and moderate‐to‐severe asthma (*n* = 32) defined using the Global Initiative For Asthma (GINA) guidelines.^1^ Additional bronchial tissue was obtained from asthmatic subjects and nonasthmatic controls undergoing research bronchoscopies (*n* = 43) or lung resection surgery (*n* = 6) from which primary ASM cells/bronchial tissue sections could be derived. The use of lung resection tissue was approved by the National Research Ethics Service (07/MRE08/42). The use of biopsies was approved by the Leicester and Northamptonshire Ethics Committee (08/H0406/189 and 05/Q2502/98). All patients gave their written informed consent. Post‐lung transplant tissue was obtained from postmortem specimens taken from recipients who died >120 days post‐transplant. Approval was given by the West Midlands Research Ethics Committee (07/H1208/47).

### Immunohistochemistry

Fibrocytes (α‐smooth muscle actin (SMA)^+^ CD34^+^ collagen I^+^ cells^9^) were enumerated in the smooth muscle bundle (α‐SMA^+^ CD34^−^ collagen I^−^ cells) in bronchial tissue embedded in glycomethacrylate (GMA, Sigma‐Aldrich)^47^ from healthy controls (*n* = 15) versus patients with NAEB (*n* = 12), COPD (*n* = 11) and asthma (*n* = 32). For each subject, sequential 2‐µm sections were cut and stained for α‐SMA using an α‐SMA antibody (clone 1A4, Dako, Agilent Technologies) or mouse immunoglobulin (Ig)G2a isotype control (Dako Agilent Technologies), cluster of differentiation (CD) 34 using a mouse monoclonal anti‐CD34 antibody (clone QBEnd‐10, Abnova, R&D systems), collagen I (clone 5D8‐G9, Millipore, Sigma‐Aldrich) and mouse IgG1 isotype control (Dako Agilent Technologies). Fibrocytes were identified as the subset of α‐SMA‐positive cells per mm^2^ ASM that also stained positive for CD34 and collagen I in sequential sections using colocalisation. Assessors were blind to clinical characteristics.

Proliferating ASM cells, identified as α‐SMA^+^‐proliferating cell nuclear antigen (PCNA)^+^ cells in sequential sections, were assessed in bronchial biopsies embedded in GMA from healthy controls (*n* = 9), asthmatics (*n* = 25) and in lung tissue from lung transplant recipients embedded in paraffin (*n* = 8) which served as a positive control for detection of PCNA^+^ cells as smooth muscle proliferation is common in lung transplant recipients. For each subject, sequential 2‐µm sections were cut and stained for α‐SMA (clone 1A4, Dako, Agilent Technologies), proliferating cell nuclear antigen (PCNA, clone PC10, Leica Biosystems) or mouse IgG2a isotype control (Dako, Agilent Technologies). Proliferating ASM cells were identified as the subset of α‐SMA‐positive cells in the ASM that also stained positive for PCNA in sequential sections using colocalisation. Assessors were blind to clinical characteristics.

Tissue sections from 1 sample per subject were stained and analysed under an Olympus BX50 light microscope (Olympus UK and Ireland). Assessors were blind to clinical characteristics.

Following enumeration, Spearman's correlations of fibrocyte number per mm^2^ ASM in asthma with age, age at diagnosis, lung function, body mass index, number of exacerbations in the last 12 months, total IgE, sputum eosinophils, FeNO and symptom scores (Asthma Control Questionnaire 6 and Asthma Quality of Life Questionnaire) were performed.

### Cell culture

In a separate cohort of subjects, ASM bundles were isolated from bronchial biopsies (*n* = 28 asthmatic, *n* = 15 nonasthmatic) and lung resection material (*n* = 6, nonasthmatic). Primary ASM cells were cultured in Dulbecco's modified Eagle's medium (DMEM) with GlutaMAX‐1 (Gibco, Thermo Fisher Scientific) supplemented with 10% foetal bovine serum (FBS, Gibco), 1% antibiotic–antimycotic (AA: 100U mL^−1^ penicillin, 100 μg mL^−1^ streptomycin, 0.25 μg mL^−1^ amphotericin, Gibco), 1% nonessential amino acids (NEAA: 100 μM, Gibco) and 1% sodium pyruvate (SP: 1 mM) (Sigma‐Aldrich). Cells were characterised for α‐SMA expression using a mouse monoclonal anti‐α‐SMA antibody (clone 1A4, Dako, Agilent Technologies) or mouse IgG2a isotype control (Dako, Agilent Technologies) by flow cytometry and used between passages 2 and 6.

Fibrocytes were isolated from peripheral blood mononuclear cells (PBMCs) from nonasthmatics and asthmatics using a previously validated procedure.^9^ PBMCs were isolated from peripheral blood using Histopaque (Sigma‐Aldrich) density gradient centrifugation, washed twice with Hank's buffered saline solution (HBSS, Gibco) and cultured overnight in DMEM with 10% FBS, 1% AA, 1% NEAA and 1% SP in tissue culture flasks coated with 40 µg mL^−1^ fibronectin (Sigma‐Aldrich). Nonadherent cells were then removed, and cells were then cultured in DMEM with 10% FBS, 1% AA, 1% NEAA and 1% SP for a further 5–10 days prior to experimentation, until development of spindle‐like morphology characteristic of fibrocytes. The number of contaminating T cells was assessed with a haemocytometer and was routinely < 10%. Blood was collected from nonasthmatic and asthmatic volunteers under ethical approval (09/H0402/4 and 08/H0406/189, approved by Leicester and Northamptonshire Ethics Committee). All subjects gave written informed consent.

For peripheral blood‐derived fibrocyte/ASM co‐culture, tissue culture (TC)‐coated dishes (Thermo Fisher Scientific) were incubated with 10 µg mL^−1^ fibronectin in phosphate‐buffered saline (PBS, Sigma‐Aldrich) for at least 30 min at 37ºC; dishes were then washed with PBS prior to addition of ASM cells/media alone (for fibrocyte mono‐cultures). ASM cells were seeded onto 60‐mm‐ or 100‐mm‐diameter TC dishes coated with 10 µg mL^−1^ fibronectin at a density of 2–6 × 10^5^ cells per dish, respectively, and allowed to adhere overnight prior to addition of carboxyfluorescein succinimidyl ester (CFSE, Thermo Fisher Scientific)‐labelled fibrocytes. Fibrocytes were labelled with 5 µM CFSE in PBS, according to manufacturer's instructions, prior to subsequent mono‐culture or co‐culture with ASM cells, at a density of 1–3 × 10^5^ cells per 60‐mm‐ or 100‐mm‐diameter TC dish, respectively, for 3–4 or 7–8 days. A previous study has shown 2 ASM cells: 1 fibrocyte to be the optimum ratio for detecting changes following co‐culture.^19^ Where appropriate, cultures were incubated with 100 ng mL^−1^ interleukin (IL)‐6 ± 10 µg mL^−1^ anti‐IL‐6, clone 6708 or mIgG1 (R&D systems), IL‐4 and IL‐13 (both 10 ng mL^−1^, R&D systems). Cells were harvested using trypsin (Gibco) and used in experiments detailed below. For each co‐culture experiment, the number of experiments (n, i.e. the number of different combinations of fibrocyte and ASM donors) is given followed by the number of different fibrocyte and ASM donors used (number of fibrocyte donors and number of ASM donors). Some fibrocyte donors were co‐cultured with more than one ASM donor, and some ASM donors were co‐cultured with more than one fibrocyte donor. Fibrocytes were also incubated with C‐C motif chemokine (CCL)11, CCL19, C‐X‐C motif chemokine (CXCL)10, chemokine (C‐X3‐C motif) ligand 1 (CX3CL1), IL‐8, IL‐1β, tumor necrosis factor (TNF)‐α, stromal cell‐derived factor (SDF)‐1α and CCL2 prior to measurement of CD14 expression (all R&D systems).

### Flow cytometry

Following co‐culture, cells were fixed with phosphate‐buffered saline (PBS) containing 4% paraformaldehyde (Sigma) followed by incubation with primary antibodies against the protein of interest (CD14, clone TUK4, Dako; TGFβRI, Abcam; ICAM‐1, clone BH17, Abcam) versus appropriate isotype controls (mIgG2a, Dako; mIgG1, Dako; and rbIgG, Cell Signaling Technology, Leiden, Netherlands) or fixed and permeabilised with PBS containing 4% paraformaldehyde and 0.1% saponin (Sigma), followed by incubation with primary antibodies against the protein of interest (α‐SMA, clone 1A4, Dako; h‐Caldesmon, clone hHCD, Sigma; SM‐MHC, clone SMMS‐1, Dako) versus appropriate isotype controls (mIgG2a, Dako, and mIgG1, Dako) and subsequent incubation with goat anti‐mouse or goat anti‐rabbit allophycocyanin (APC)‐conjugated secondary antibodies (Invitrogen, Thermo Fisher Scientific) in PBS containing 0.5% BSA ± 0.1% saponin. APC secondary antibodies were used as the fluorescence spectra do not overlap with that of CFSE. Flow cytometry was performed with a FACSCanto (BD Biosciences) and analysis performed with FlowJo vX software (FlowJo LLC). By plotting FSC against SSC, the overlapping ASM and fibrocyte populations were identified and contaminating T‐cell population gated out from subsequent analysis. ASM and fibrocyte SSC were then plotted against CFSE fluorescence allowing fibrocytes and ASM to be gated separately to examine protein expression and to ascertain the ratio of CFSE‐positive fibrocytes:unlabelled ASM for assessment of ASM cell number post co‐culture. Cells were gated for analysis as shown in Supplementary figure 5a and b, with the right‐hand panel of Supplementary figure 5b showing the distinction between the CFSE‐labelled fibrocyte population (green) and CFSE‐free ASM cells (identified using DAPI staining of nuclei). In addition, the geometric mean forward (in the direction of the laser path) and side (at 90 degrees to the laser path) light scatter characteristics (FSC and SSC, respectively) of the fibrocyte and ASM populations were calculated as a measure of cell size and cell granularity, respectively, to give insight into the phenotypic complexity, the apoptotic/necrotic status and hypertrophic status of cells following fibrocyte/ASM co‐culture or between nonasthma and asthma.

### Immunofluorescence

ASM cells were seeded into 8‐well chamber slides coated with 10 µg mL^−1^ fibronectin at a density of 1 × 10^4^ allowed to adhere overnight. ASM cells were then co‐cultured with 0.5 × 10^4^ CFSE‐labelled fibrocytes for 7–8 days. Apoptosis was induced with 0.1 µM staurosporine (STS, Sigma) 20h prior to fixation with methanol and staining of cell nuclei with 4′,6‐diamidino‐2‐phenylindole (DAPI, Sigma). Nuclei of unlabelled ASM cells were assessed for uniform staining with clear margins (nonapoptotic) or fragmented bright staining of condensed nuclear material (apoptotic). An Olympus BX50 fluorescent microscope mounted with an Olympus DP72 camera was used to visualise staining.

### ELISA

Following co‐culture, cell‐free SNs from fibrocyte/ASM co‐cultures and respective fibrocyte and ASM mono‐cultures were collected. TGF‐β1 (R&D systems) and IL‐6 (R&D systems) were measured by ELISA according to manufacturer's instructions. Each sample was measured in duplicate.

### Collagen gel contraction assay

The collagen gel contraction assay was performed with the following: (i) fibrocytes/ASM co‐cultured for 3–4 days (flow cytometry was used to calculate ASM cell number post co‐culture, as described above to ensure addition of equivalent numbers of ASM cells to each collagen gel), (ii) ASM following co‐culture with fibrocytes seeded onto Transwells for 3–4 days, (iii) ASM cultured alone with contraction stimulated by bradykinin in the presence of media alone, fibrocyte/ASM co‐culture SNs and the respective ASM mono‐culture SNs and (iv) ASM and fibrocytes added to the collagen gel mixture together with no prior co‐culture at the range of fibrocyte‐to‐ASM ratios present following co‐culture. In each case, the collagen gel mixture (299 µL PureCol, 3 mg mL^−1^ (Cell Systems, Troisdorf, Germany), 37 µL 10× DMEM (Gibco), 20 mL sodium bicarbonate, 7.5% (Gibco)) was impregnated with 1.25 × 10^5^ ASM cells ± fibrocytes (in 144 µL of serum‐free DMEM (Gibco)) as above and allowed to set for 90 min at 37°C, as described previously.^7^ Gels were detached and bradykinin (1 ng mL^−1^, Sigma) added to induce contraction. Each condition was performed in duplicate. Images were captured over a 3‐h period and gel area was measured as a percentage of well area using ImageJ software (National Institutes of Health, Bethesda, Maryland, USA) by a blinded observer, with contraction indicated by a reduction in gel size as a percentage of well area.

### Statistics

Statistical analysis was performed using GraphPad Prism (GraphPad Software). Data were tested for normality using the Shapiro–Wilk test. For normally distributed data, two‐tailed unpaired or paired *t*‐tests, one‐sample *t*‐tests or one‐way ANOVA was used as appropriate. For nonparametric data, Mann–Whitney *U*‐tests, Wilcoxon matched‐pairs signed‐rank tests or Kruskal–Wallis tests with Dunn's multiple comparison post‐test was used as appropriate. Correlations were performed using Pearson's correlation. *P* < 0.05 was considered statistically significant.

## Conflicts of Interest

CEB serves on advisory boards for GlaxoSmithKline, AstraZeneca, Boehringer Ingelheim, Chiesi and Roche; receives honoraria from Novartis; receives research support from GlaxoSmithKline, AstraZeneca, Chiesi, Novartis, Boehringer Ingelheim and Roche; and has received grants from Asthma UK, NIHR and Wellcome Trust. SHS has performed advisory services for/received speaker fees from AZ, GSK, Novartis, Roche, Chiesi, Boehringer Ingelheim, Mundipharma and Owlstone Medical; has received other fees from ERT Medical; and has received grants from NIHR. DD has received speaker fees from Chiesi, AstraZeneca and Boehringer Ingelheim. RS has received grant support from Asthma UK. DK, RB, LC and RDT have no conflicts of interest.

## Author Contributions

**Ruth Saunders:** Conceptualization; Formal analysis; Funding acquisition; Investigation; Methodology; Project administration; Visualization; Writing – original draft; Writing – review and editing. **Davinder Kaur:** Conceptualization; Methodology; Writing – review and editing. **Dhananjay Desai:** Formal analysis; Investigation; Visualization; Writing – review and editing. **Rachid Berair:** Investigation; Visualization; Writing – review and editing. **Latifa Chachi:** Investigation; Visualization; Writing – review and editing. **Richard D Thompson:** Conceptualization; Writing – review and editing. **Salman H Siddiqui:** Conceptualization; Funding acquisition; Writing – review and editing. **Christopher E Brightling:** Conceptualization; Funding acquisition; Supervision; Writing – review and editing.
