# Supplementary material for: Fibrocyte localisation to the ASM bundle in asthma: bidirectional effects on cell phenotype and behaviour
Source: Clin Transl Immunology. 2020 Nov 13;9(11):e1205. doi: 10.1002/cti2.1205 (PMC7662089; doi:10.1002/cti2.1205)

Supplementary material

Fibrocyte localisation to the ASM-bundle in asthma: Bi-directional effects on cell phenotype and behaviour

Running title: Airway smooth muscle-fibrocyte interactions

Ruth Saunders[1], Davinder Kaur[1], Dhananjay Desai[1§], Rachid Berair[1^], Latifa Chachi[1], Richard D Thompson[2], Salman H Siddiqui[1], Christopher E Brightling[1].

[1]Institute for Lung Health, Department of Respiratory Sciences, University of Leicester, UK, [2]University Hospitals Birmingham NHS Foundation Trust, Birmingham, UK

Corresponding author: Dr Ruth Saunders, Department of Respiratory Sciences, Clinical Sciences Wing, University Hospitals of Leicester NHS Trust, Glenfield Hospital, Groby Road, Leicester, LE3 9QP. Tel: +44 (0) 116 258 3011, email: rms4@le.ac.uk

[§] currently at University Hospitals Coventry & Warwickshire NHS Trust, Coventry, UK

[^] currently at The Royal Wolverhampton NHS Trust, Wolverhampton, UK

Supplementary figure 1. Fibrocyte numbers are increased in the ASM in late onset vs early onset asthma. **(a)** Fibrocyte numbers $mm^{2\ -1}$ ASM correlate with age of onset of asthma, n = 32, Spearman's correlation, r = 0.351, *P* = 0.049. **(b)** Fibrocyte numbers $mm^{2\ -1}$ ASM are increased in late onset (n = 17) versus early onset asthma (n = 15), *P* = 0.040, Mann-Whitney *U*-test. Data expressed as mean ± sem.

Supplementary figure 2. h-Caldesmon and MHC expression in fibrocytes following co-culture with ASM from asthmatics compared to non-asthmatics. **(a)** Change in percentage of fibrocytes expressing h-caldesmon following co-culture with ASM from non-asthmatics (n = 6 different co-culture experiments (with fibrocytes from 5 different donors co-cultured with ASM from 4 different donors), * *P* = 0.043) or asthmatics (n = 8 different co-culture experiments (with fibrocytes from 7 different donors co-cultured with ASM from 5 different donors), *P* = 0.950) versus respective mono-cultures (two-tailed paired *t*-tests). **(b)** Change in percentage of fibrocytes expressing MHC following co-culture with ASM from non-asthmatics (n = 7 different co-culture experiments (with fibrocytes from 7 different donors co-cultured with ASM from 5 different donors), *P* = 0.054) or asthmatics (n = 6 different co-culture experiments (with fibrocytes from 5 different donors co-cultured with ASM from 5 different donors), *P* = 0.105), versus respective mono-cultures (two-tailed paired *t*-tests). *P*-values on graphs represent comparison of expression levels (expressed as percentage change versus respective FC mono-cultures) in fibrocytes following co-culture with ASM from non-asthmatics versus asthmatics (two-tailed unpaired *t*-tests). Data are expressed as mean ± sem.

Supplementary figure 3. Expression of smooth muscle differentiation related proteins following fibrocyte/ASM co-culture. **(a)** ELISA was used to compare the sum of TGF-β1 present in culture SNs from fibrocyte plus ASM mono-cultures versus the respective co-cultures of fibrocytes with ASM from non-asthmatics (n = 11 different co-culture experiments

(with fibrocytes from 10 different donors co-cultured with ASM from 6 different donors), *P* = 0.277) and asthmatics (n = 12 different co-culture experiments (with fibrocytes from 10 different donors co-cultured with ASM from 9 different donors), *P* = 0.291. **(b)** Change in percentage of ASM from non-asthmatics (n = 10 different co-culture experiments (with fibrocytes from 9 different donors co-cultured with ASM from 6 different donors), *P* = 0.531) or asthmatics (n = 16 different co-culture experiments (with fibrocytes from 14 different donors co-cultured with ASM from 10 different donors),, *P* = 0.479) expressing α-SMA (Wilcoxon matched pairs signed rank tests). **(c)** change in percentage of ASM from non-asthmatics (n = 6 different co-culture experiments (with fibrocytes from 5 different donors co-cultured with ASM from 4 different donors), *P* = 0.725), or asthmatics (n = 8 different co-culture experiments (with fibrocytes from 7 different donors co-cultured with ASM from 5 different donors), *P* = 0.879) expressing h-caldesmon (two-tailed paired *t*-tests). **(d)** change in percentage of ASM from non-asthmatics (n = 7 different co-culture experiments (with fibrocytes from 7 different donors co-cultured with ASM from 5 different donors), *P* = 0.449) or asthmatics (n = 6 different co-culture experiments (with fibrocytes from 5 different donors co-cultured with ASM from 5 different donors), *P* = 0.766) expressing MHC (two-tailed paired *t*-tests). **(e)** change in percentage of ASM from non-asthmatics (n = 10 different co-culture experiments (with fibrocytes from 10 different donors co-cultured with ASM from 6 different donors), * *P* = 0.015) or asthmatics (n = 13 different co-culture experiments (with fibrocytes from 11 different donors co-cultured with ASM from 9 different donors) n = 13 (11, 9), *P* = 0.232) expressing TGFβR1 (two-tailed paired *t*-tests) following co-culture with fibrocytes versus respective mono-cultures. For **(b, c, d and e)**: *P*-values on graphs represent comparison of expression levels (expressed as percentage change versus respective ASM mono-cultures) between non-asthmatic and asthmatic ASM following co-culture with fibrocytes (**(b)** Mann-Whitney *U*-test, **(c, d and e)**: two-tailed unpaired *t*-tests). **(f)** The percentage of fibrocytes (n = 3 different co-

culture experiments (with fibrocytes from 3 different donors co-cultured with ASM from 3 different donors), P = 0.250, Wilcoxon matched pairs signed rank test) and ASM (n = 3 different co-culture experiments (with fibrocytes from 3 different donors co-cultured with ASM from 3 different donors), P = 0.001, paired t-test) expressing ICAM-1 in mono- vs co-culture. Data are presented as mean ± sem.

Supplementary figure 4. Addition of independently cultured fibrocytes and ASM to collagen gels or SNs from fibrocyte/ASM co-cultures does not result in enhanced bradykinin-stimulated collagen gel contraction. **(a)** Time-course, **(b)** $AUC_{180mins}$ and **(c)** example photomicrographs of bradykinin-stimulated contraction of collagen gels impregnated with ASM and incubated with culture media (ASM alone), and SNs from ASM cultured alone or co-cultured with fibrocytes for 3-4 days (n = 7 different co-culture experiments (with fibrocytes from 5 different donors co-cultured with ASM from 5 different donors), P = 0.871; one-way ANOVA). **(d)** Time-course, **(e)** $AUC_{180mins}$ and **(f)** example photomicrographs of bradykinin-stimulated contraction of collagen gels impregnated with independently cultured ASM and fibrocytes corresponding to the range of fibrocyte:ASM (FC:ASM) ratios (1:4 to 1:32) present in 3-4 day co-cultures compared to ASM alone (n = 9 different co-culture experiments (with fibrocytes from 8 different donors co-cultured with ASM from 8 different donors), P = 0.990; one-way ANOVA). Data are plotted as mean ± sem.

Supplementary figure 5. Strategy for distinguishing fibrocytes from ASM by flow cytometry following co-culture. **(a)** Fibrocyte and ASM FSC and SSC characteristics overlap (left-hand panel), CFSE labelling and subsequent plotting of SSC versus CFSE fluorescence allows distinction between the unlabelled $CFSE^{low}$ ASM population and the labelled $CFSE^{high}$ fibrocyte population (middle panel) and subsequent gating for analysis. The right hand panel

shows $DAPI^+$ $CFSE^+$ fibrocytes in co-culture with $DAPI^+$ $CFSE^-$ ASM cells. **(b)** SSC versus CFSE fluorescence dot plot of unlabelled ASM cells in mono-culture. **(c)** SSC versus CFSE fluorescence dot plot of CFSE labelled fibrocytes in mono-culture.

Supplementary figure 1

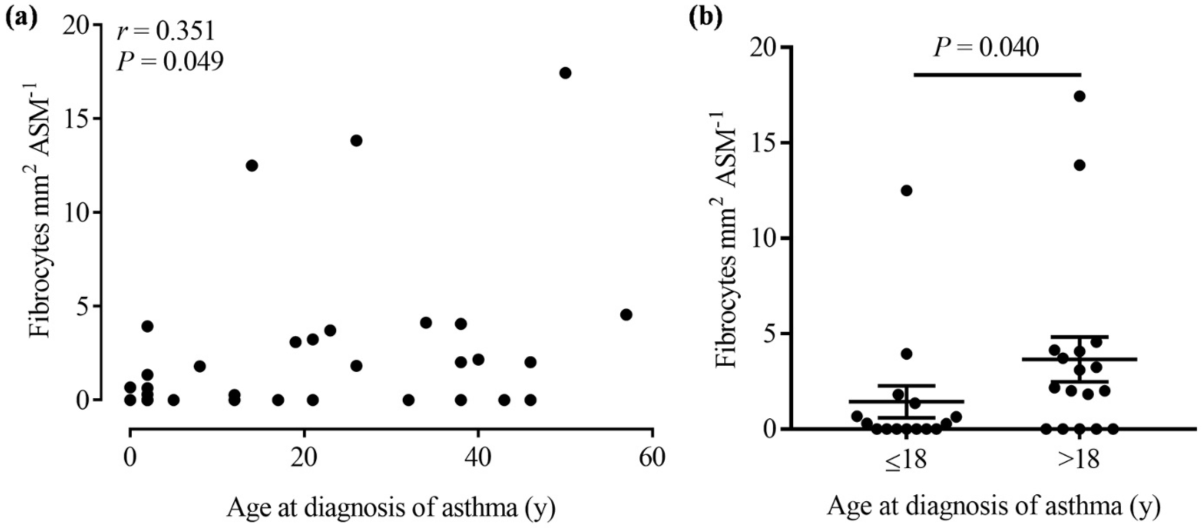

Supplementary figure 2

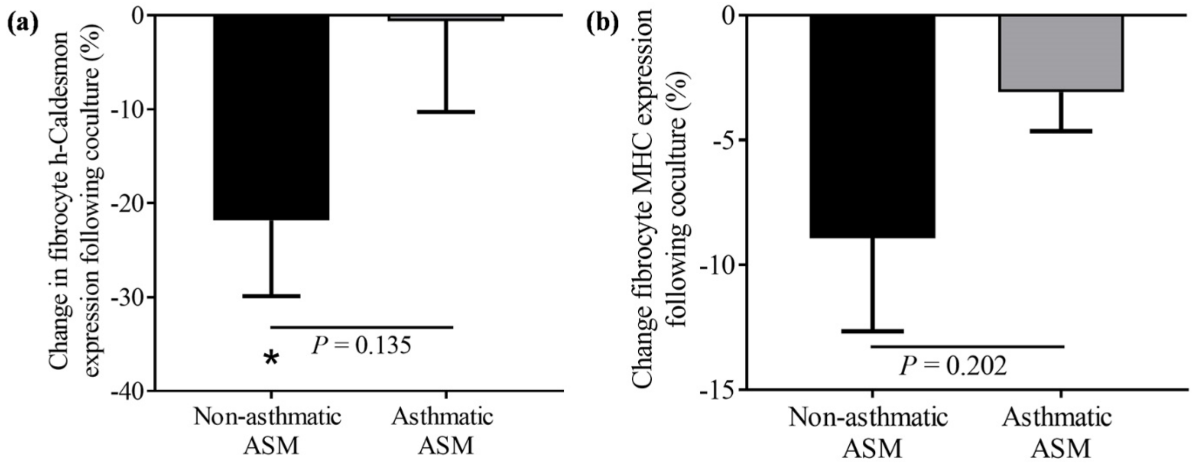

Supplementary figure 3

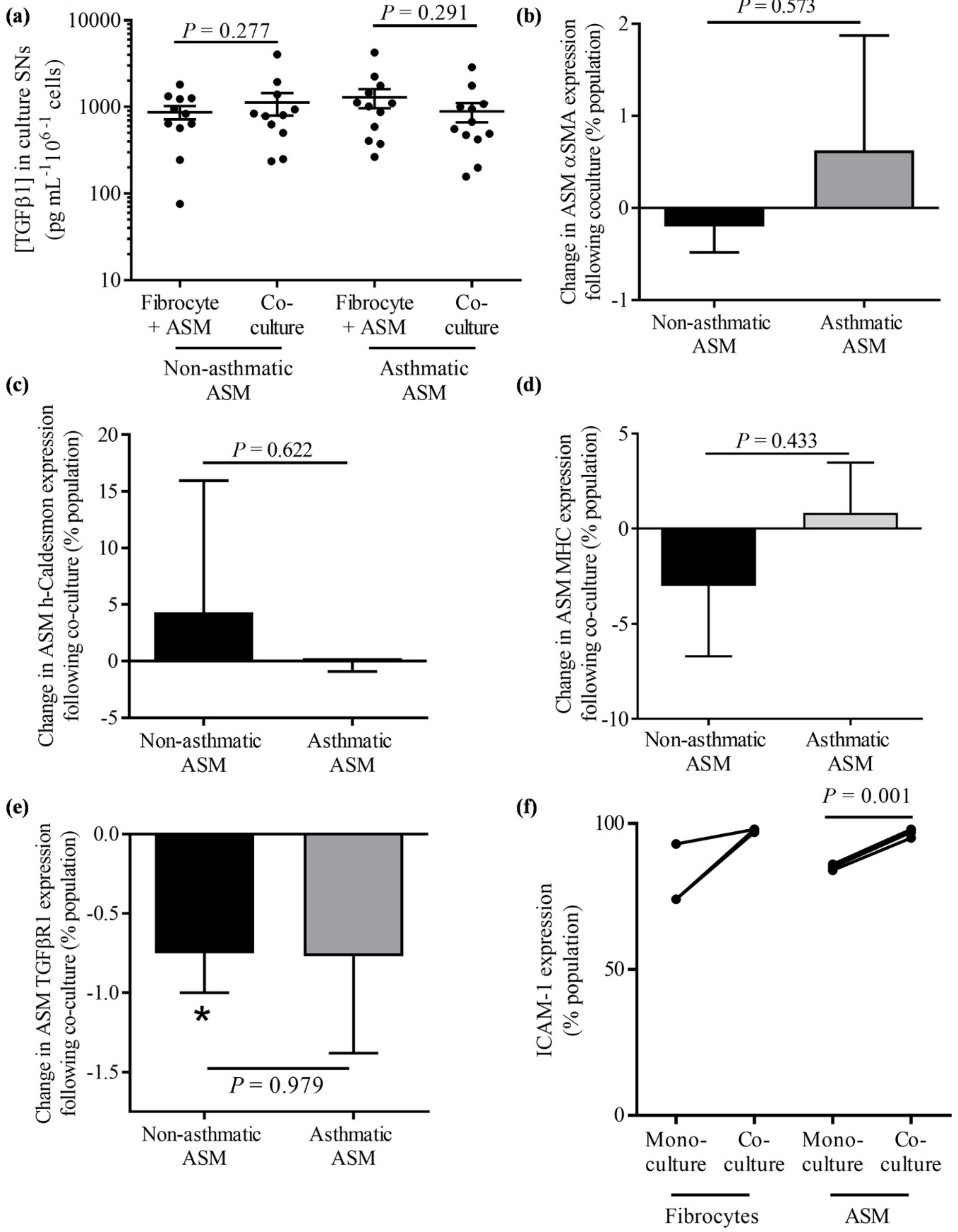

Supplementary figure 4

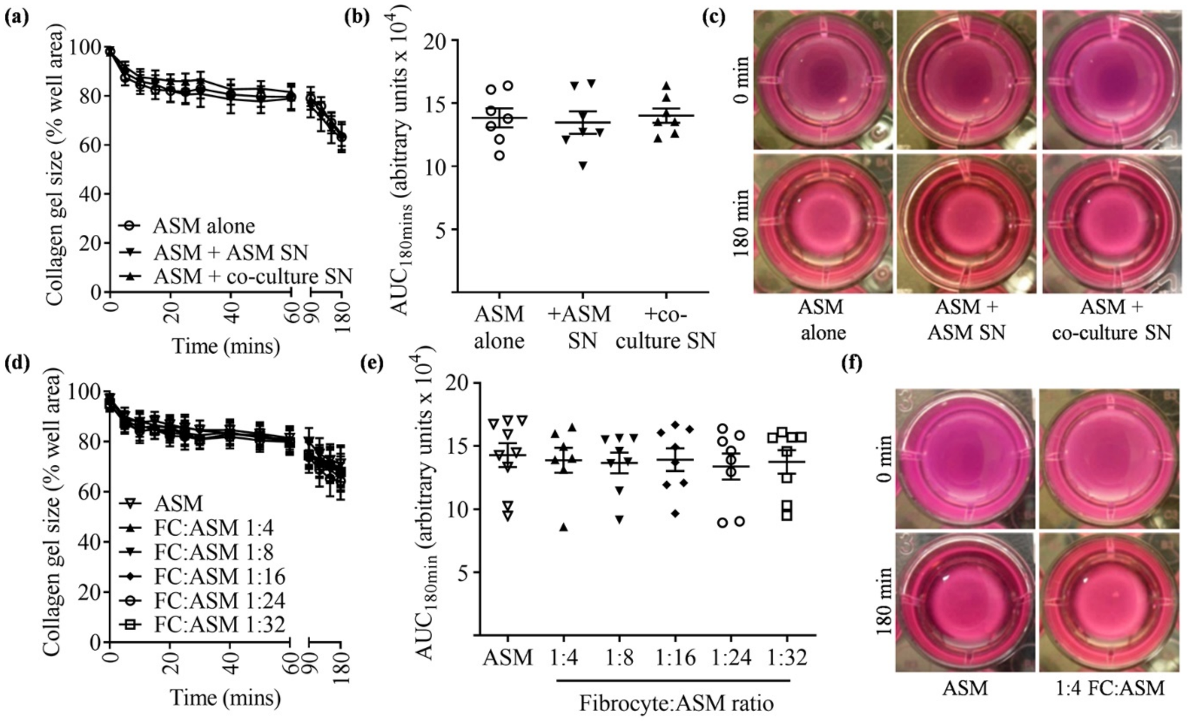

Supplementary figure 5

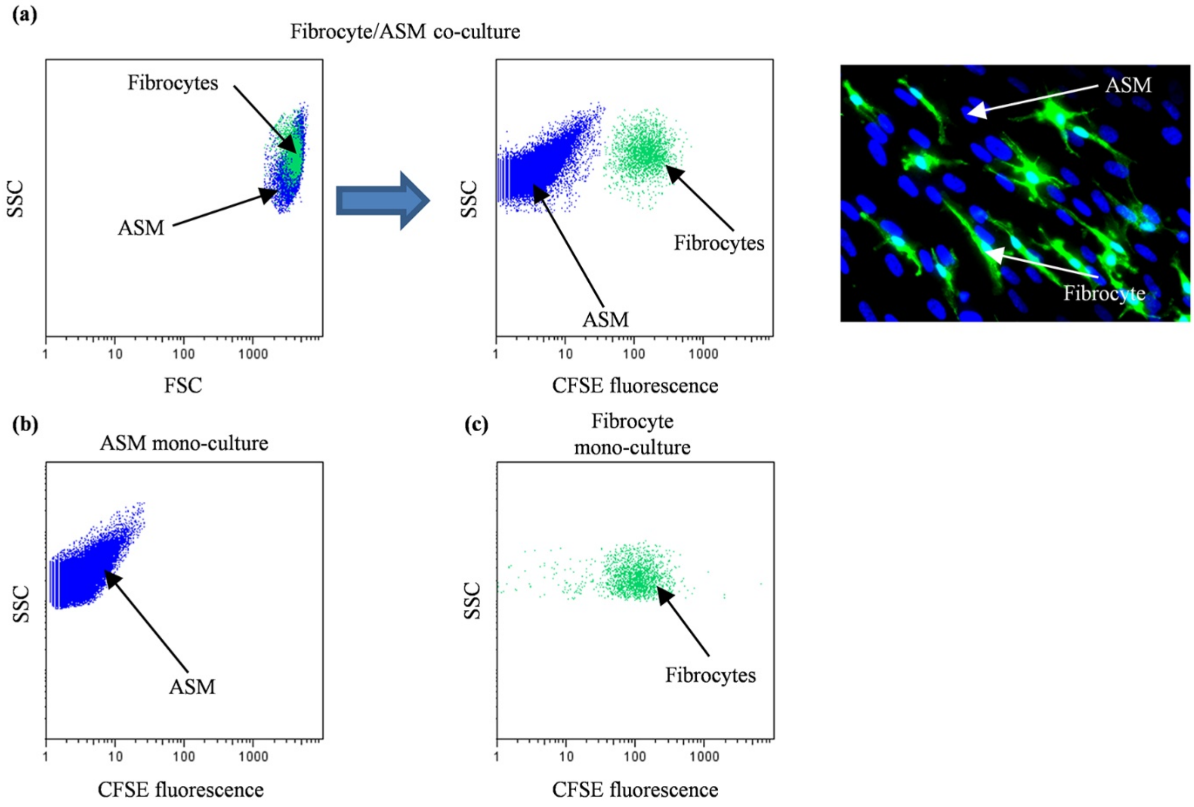

Supplement: Supplementary file 1 [file CTI2-9-e1205-s001.pdf]
